# Personalized genealogical history of UK individuals inferred from biobank-scale IBD segments

**DOI:** 10.1186/s12915-021-00964-y

**Published:** 2021-02-16

**Authors:** Ardalan Naseri, Kecong Tang, Xin Geng, Junjie Shi, Jing Zhang, Pramesh Shakya, Xiaoming Liu, Shaojie Zhang, Degui Zhi

**Affiliations:** 1 School of Biomedical Informatics, The University of Texas Health Science Center at Houston, Houston, TX 77030 USA; 2 Department of Computer Science, University of Central Florida, Orlando, FL 32816 USA; 3 USF Genomics, College of Public Health, University of South Florida, Tampa, FL 33612 USA; 4 Center for Precision Health, School of Biomedical Informatics, School of Public Health, The University of Texas Health Science Center at Houston, Houston, TX 77030 USA

**Keywords:** Identity by descent, RaPID, UK Biobank, Genealogical history

## Abstract

**Background:**

The genealogical histories of individuals within populations are of interest to studies aiming both to uncover detailed pedigree information and overall quantitative population demographic histories. However, the analysis of quantitative details of individual genealogical histories has faced challenges from incomplete available pedigree records and an absence of objective and quantitative details in pedigree information. Although complete pedigree information for most individuals is difficult to track beyond a few generations, it is possible to describe a person’s genealogical history using their genetic relatives revealed by identity by descent (IBD) segments—long genomic segments shared by two individuals within a population, which are identical due to inheritance from common ancestors. When modern biobanks collect genotype information for a significant fraction of a population, dense genetic connections of a person can be traced using such IBD segments, offering opportunities to characterize individuals in the context of the underlying populations. Here, we conducted an individual-centric analysis of IBD segments among the UK Biobank participants that represent 0.7% of the UK population.

**Results:**

We made a high-quality call set of IBD segments over 5 cM among all 500,000 UK Biobank participants. On average, one UK individual shares IBD segments with 14,000 UK Biobank participants, which we refer to as “relatives.” Using these segments, approximately 80% of a person’s genome can be imputed. We subsequently propose genealogical descriptors based on the genetic connections of relative cohorts of individuals sharing at least one IBD segment and show that such descriptors offer important information about one’s genetic makeup, personal genealogical history, and social behavior. Through analysis of relative counts sharing segments at different lengths, we identified a group, potentially British Jews, who has a distinct pattern of familial expansion history. Finally, using the enrichment of relatives in one’s neighborhood, we identified regional variations of personal preference favoring living closer to one’s extended families.

**Conclusions:**

Our analysis revealed genetic makeup, personal genealogical history, and social behaviors at the population scale, opening possibilities for further studies of individual’s genetic connections in biobank data.

**Supplementary Information:**

The online version contains supplementary material available at 10.1186/s12915-021-00964-y.

## Background

An individual’s genealogical history is often of interest to the innate curiosity about one’s ancestry. However, it has been challenging to collect the complete and accurate family pedigree of an individual. Further, the pedigree record is often incomplete and lacking objective, quantitative details of one’s ancestors and relatives. As a result, population studies of personal genealogical history are often void of quantitative rigor.

Thanks to the establishment of modern biobanks and direct-to-consumer (DTC) genetic testing companies which contain genotype information of a relatively large fraction (0.1–5%) of a population, it is now possible to identify dense genetic connections, as manifested by identical-by-descent (IBD) segments, among individuals. IBD segments are shared DNA segments between two individuals that are inherited from a common ancestor. This opens the possibility to study the quantitative details of one’s genealogical history and identify emerging patterns.

The patterns of IBD segments in large cohorts and biobanks have been extensively studied. However, the scope of some studies is limited to unveil detailed pedigree information [1]. Other IBD segment analyses aim to reveal overall population demographic histories [2–6] and clusters [7]. There has been a lack of studies that investigate personal genealogical history at an intermediate scale. Although the complete pedigree for most individuals is difficult to trace beyond a few generations, it is possible to describe a person’s genealogical history using their genetic relatives revealed by IBD segments.

Here, we present a comprehensive analysis of the personal genealogical history from all identity-by-descent (IBD) segments of an individual in a biobank of a large modern population. We first made a high-quality call set of IBD segments over 5 cM among all 500,000 UK Biobank [8] participants. Based upon the genetic connections revealed through these IBD segments, we investigate the “relative cohort,” all persons who share at least one IBD segment, of individuals. We discuss a set of genealogical descriptors (GPs) based on the relative cohort and show that these genealogical descriptors offer very rich information about one’s genetic makeup, personal genealogical history, and social behaviors.

## Results

### IBD segment calling and quality assessment

Using RaPID [9], an efficient and accurate method, we identified 3.5 billion IBD segments over 5 cM within the 22 autosomes among 487,409 UK Biobank participants (Methods: the “IBD segment calling using RaPID” section). Thanks to the efficiency of RaPID, we achieved this in 5.25 days using a single-core CPU with 6.34G peak memory. This translates to, on average, each person having 5 cM genetic connections to 14,000, or about 3% of UKBB persons, whom we call genetic relatives or relatives for short. These segments offer on average 10x coverage of the overall diploid genome for a UK individual.

Noting that this density of IBD segments is higher than that reported by existing studies [10], we took extra caution assessing the quality of our results. Since quality assessment of IBD segment calls of a large cohort is a less-studied problem, we developed a strategy for evaluating IBD segment calls in an “unsupervised” fashion: First, we compared the kinship coefficients derived from RaPID’s IBD calls against a standard genotype-based relatedness caller, KING [11]. We verified that RaPID’s call is indeed consistent with the theoretical expectation for close relatives (Additional file 1: Fig. S1).

Second, we verified the consistency of RaPID’s calls with traditional methods. As not all methods can run to call > 5 cM IBD segments for all UK Biobank participants without extravagant computing resources, we used a subset of 200,000 participants and ran over chromosome 22. We ran traditional methods including GERMLINE [12], Refined IBD [13], and also a recent method iLASH [14], and calculated the overlap between these sets of IBD calls. We found that RaPID’s results included almost all segments called by other methods: 95.8% of GERMLINE’s calls, 97.2% of iLash’s calls, and 99.4% of Refined IBD’s calls (Additional file 1: Table S1 and Fig. S2). Interestingly, RaPID detected 16.3% more segments that were missed by all other methods.

Third, we leveraged the monozygotic (MZ) twin pairs to estimate the detection power of IBD segments. While MZ twins should match their entire chromosomes, in real data, the long IBD segments are interrupted by switch errors in phasing. Fortunately, these MZ twins are easy to call from non-IBD calling methods, such as KING. Using the 179 twin pairs identified by KING, we observe that using the average haplotype matching identity over the moving average of windows with 100 SNPs, the switch errors are notably visible (Additional file 1: Fig. S3). Thus, these switch errors created IBD segments of various lengths. A perfect IBD segment detector should identify multiple segments that in aggregate cover the entire length of the genome.

We define the power of a method over one sample as the percent of the sites over the entire genome that was covered by any segment detected by the method. The average power values over all twin pairs of these methods are shown in Additional file 1: Table S2 and Fig. S4. RaPID has consistently demonstrated higher power when compared to other methods. It is also of interest that the power values of all methods between British twin pairs are higher, probably due, in part, to the fact that British individuals have superior genotyping and phasing quality. Overall, RaPID’s power for detecting IBD segments among MZ twins is approximately 1% higher than GERMLINE for British and 5% higher for non-British. The aforementioned benchmark may not generalize to the detection power across all pairs of individuals in UK Biobank since the MZ twin haplotypes contain probably fewer phasing errors. Therefore, the results represent a lower bound for loss of detection power.

Fourth, we leveraged parent/offspring pairs to evaluate the accuracy of IBD calling. Overall, each method achieved an accuracy of around 99.9% (see Additional file 1: Table S3), as there is very little chance that a false positive segment over 5 cM is called by any of these methods. We also evaluated the consistency of reported IBD segments using trio information similar to the study in [15]. We used the average parent-coverage overlap rate, defined as the average of the percent of child-other segments that are also detected in at least one of the parent-other segments. The average parent-coverage overlap rate of RaPID is 98.51% which is in line with other tools. Interestingly, the average parent-coverage overlap rate for the RaPID segments shared with any other tool is 95.92% (see Additional file 1: Table S4). Including unique RaPID results will contribute to an even higher consistency of shared IBDs among trios and others. RaPID segments have on average a higher mismatch rate which may indicate that some of the unique segments might have been inherited from a more distant common ancestor or may contain higher genotyping errors. There is also a possibility that some of the segments may be false positives which has been addressed by conducting additional sensitivity analyses using filtered IBD segments. We also removed the parents and re-phased the UK Biobank data using SHAPEIT3 [16]. There were no major differences between the trio consistency results after rephasing the data (see Additional file 1: Table S5).

The quality of IBD calls made by RaPID is high according to the aforementioned quality assessment. We, thus, proceeded to the downstream analysis of the IBD calls. Still, out of an abundance of caution, we performed additional sensitivity analyses by excluding the segments with higher than *μ* + 2*σ* mismatch rate (2 standard deviations above the mean of all segments across the genome), retaining about 95% of segments with an average mismatch rate similar to other tools (0.055%) (Additional file 1: Table S4).

### Relative count distribution by UK regions (county)

**Fig. 1:**
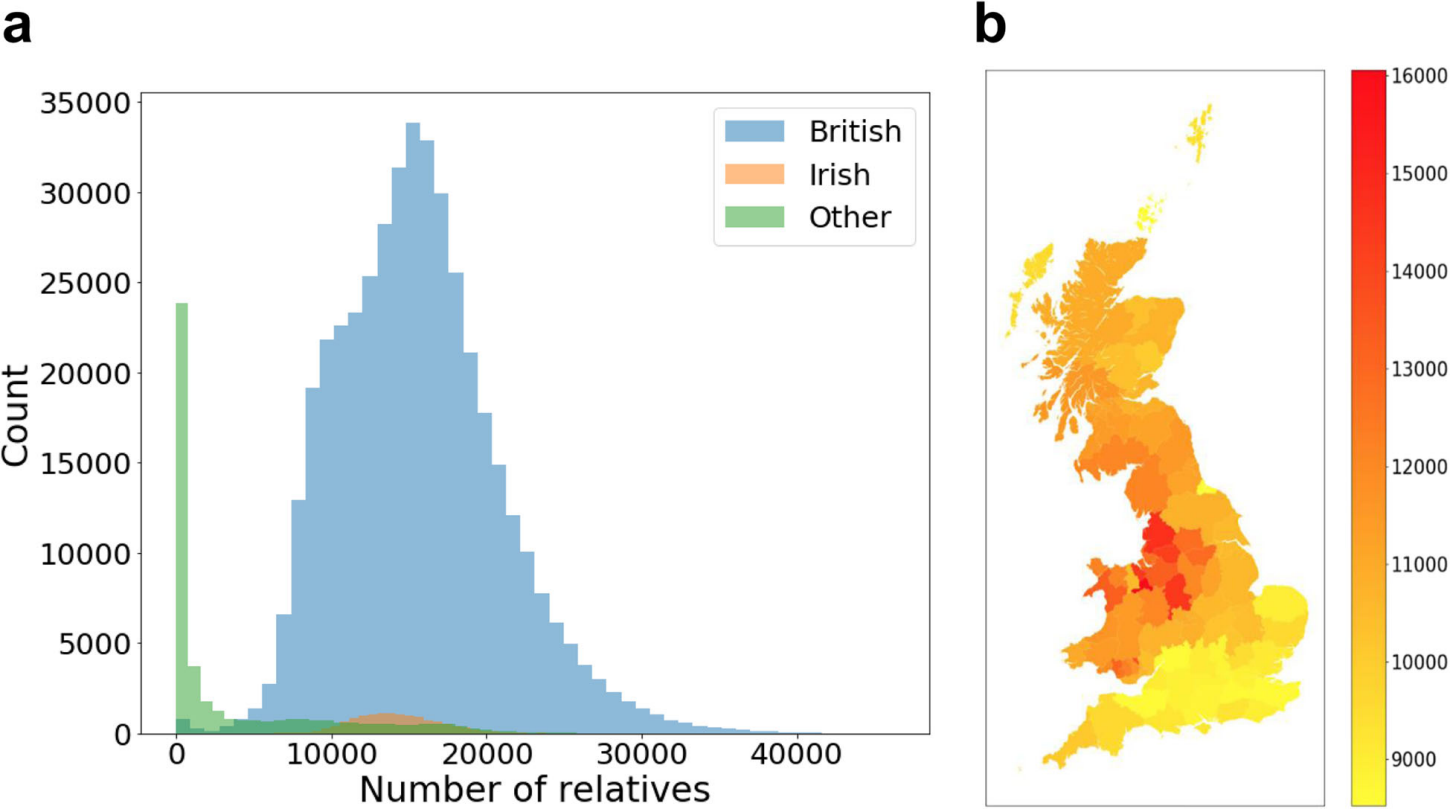
Relative count distribution (unadjusted stacked histograms of British, Irish, and others). Other refers to any other self-reported ethnic background including white. **a** Overall distribution over all individuals of all ethnicities. **b** Average relative counts of all UK areas (except Northern Ireland as home locations in Northern Ireland were not available)

Overall, average kinship coefficients across self-reported ethnic backgrounds are consistent with expectations (Additional file 1: Fig. S5). The distribution of relative counts of all UKBB participants is shown in Fig. 1a. Two individuals were considered as relatives if they share any IBD segments with a length greater than or equal to 5 cM. While the peak with relative count < 4000 is mainly due to other ethnic backgrounds rather than British and Irish, there are two peaks noticeable for the British people (Additional file 1: Fig. S6).

We found that individuals from central England (e.g., Manchester) have almost twice as many relatives as those from southern England (e.g., London) (Fig. 1b). The two peaks persist even after adjusting the relative counts by the uneven sampling rates of different regions (Additional file 1: Fig. S6c and [17]) (Methods: the “Relative count adjustment by the regional sampling rate” section). Also, the inter-region relative counts indicate clusters that are consistent with geographic boundaries (Additional file 1: Fig. S7). It is evident that Welsh and Scottish people form relatively tight clusters, respectively. Northern England has a visible cluster but also has significant sharing with Scottish individuals. Southern England seems to be less of a cluster and shares IBD segments evenly with most other regions.

### Personalized genealogical descriptors

Using all relatives of an individual as a cohort, we can infer the personalized genealogical history of each individual. This is only possible as each participant has a sufficiently large number of relatives (estimated 14,000) on average. Besides the obvious choice of total relative counts, we developed the following three sets of genealogy descriptors (GPs): the genome coverage of IBD segments, the relative counts stratified by IBD segment length, and the relative enrichment within a neighborhood.

### 5 cM IBD segments cover the entire diploid genome of an average UK individual 10 times

**Fig. 2:**
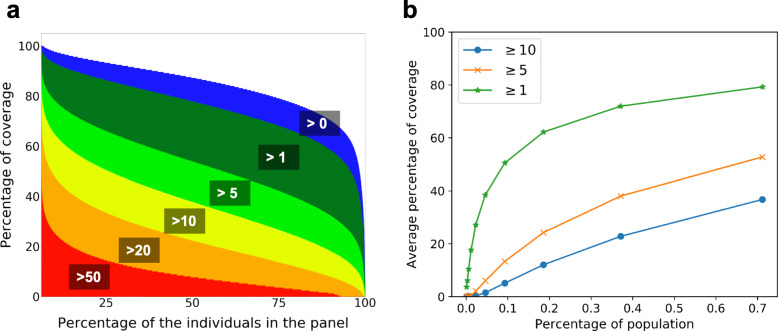
Percentage of the genome covered by ≥ 5 cM IBD segments in the UK Biobank for chromosome 1. **a** Percentage coverage of chromosome 1 by increasing the number of IBD segments, across all British individuals. **b** Average percentage coverage of chromosome 1 by IBD segments. The percentage of the population is calculated by considering the available proportion of genotyped individuals from the UK population

While 5 cM IBD segments on the average cover each individual’s genome 10 times, the coverage is highly uneven. To capture the individual variability of genome coverage, we used 5 genealogical descriptors: percent genome covered by IBD segments from 1, 2–5, 6–10, 11–20, 21–50, or larger than 50 relatives. Our analyses are focused on British people as they are well-represented (the results for all ethnicities are available in Additional file 1: Fig. S8). We found that this coverage is highly uneven among British individuals (Fig. 2a). Overall ~ 53% (229,647 out of 430,189) of the British have over 85% of their genome and ~ 73% (312,796 out of 430,189) have over 80% covered by at least 1 IBD segment. For coverage of 10 IBD segments, 48% of British individuals (205,976 out of 430,189) have over 40% covered by at least 10 segments. To an extreme, 5% of the British population (20,000 in UK Biobank) has 20% of their genome covered by over 50 segments. The coverage for a smaller population would be notably higher even using a substantially smaller panel [18, 19]. For example, it was reported that using only 128 genomes of Ashkenazi Jews, the average coverage rate was 46.7% using 5 cM segments. It was also suggested that with the availability of a panel comprising 2600 AJ or more, the coverage of genome using IBD segments would be close to 100% [18, 19].

Further, we investigated the average percent of IBD covered genomes (by any IBD segment) as a function of the percent of the population being studied (Fig. 2b). The percentage of the population is calculated by considering subsets of UK individuals divided by the total number of UK population in 2019 (67,530 K) [20]). Filtering out IBD segments with high mismatch rates also resulted in similar genome coverage (see Additional file 1: Fig. S9). The proportion of genomic bins covered by different numbers of IBD segments and the genomic coverage across chromosome 1 is shown in Additional file 1: Fig. S10. At the 0.7% sampling rate of UK Biobank, 80% of the genome is covered by at least 1 IBD segment. Even at a 0.1% sampling rate, the average genome coverage remains 50%. This fact implies a much more reliable and base-pair resolution imputation or construction of one’s genome with a very small portion of genome polymorphism (e.g., microarray genotype data) when a biobank scale database is available. Public awareness of this genome imputability is needed as to the potential issue of genome privacy (see the “Discussions” section).

### Individuals’ relative count stratified by IBD length reflects familial expansion history

**Fig. 3:**
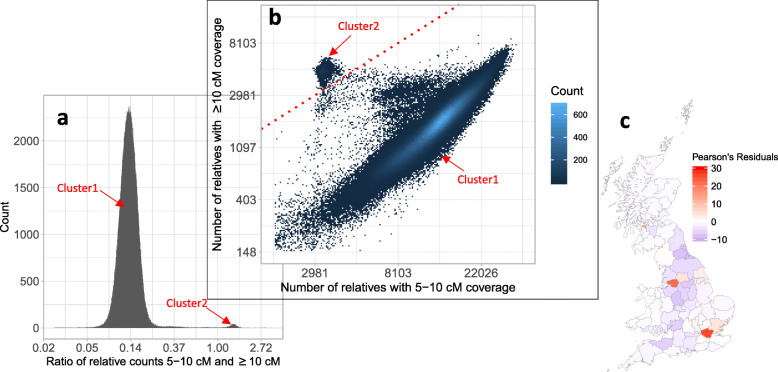
A group of 1719 individuals with distinct IBD patterns. **a** Two clusters are distinctive in the histogram of the ratio of relative counts sharing 5–10 cM and sharing 10 cM IBD segments. **b** People in cluster 2 have low relative counts in 5–10 cM segments and high relative counts in > 10 cM segments. The diagonal line is depicted as a dotted red line. **c** Regional enrichment of cluster 2

The decay pattern of relative counts with increasing IBD length reflects population history [2, 21]. Instead of analyzing the gross pattern over all individuals, we analyzed the pattern pertaining to each individual. We derived the following relative counts for each individual: the count of relatives sharing 5–10 cM, c5, and the count of relatives sharing > 10 cM segments, c10, also their ratio *c* = c10/c5. For most British individuals, the ratio is centered around 0.13. Notably, there is a small fraction of people, *n* = 1719, with *c* > 1 (Fig. 3a). Note that their c5 is among the smallest, and their c10 is among the largest (Fig. 3b). Two clusters are also still distinguishable after filtering out IBD segments with higher mismatch rates (see Additional file 1: Fig. S11). Therefore, they represent a population undergoing rapid recent expansion or represent a population with a small and constant sized population. These individuals are enriched in Greater London, Greater Manchester, and the outskirts of Glasgow (Fig. 3c). Also, their population frequency is about 0.4%. All these characteristics match that of British Jewry [22].

**Fig. 4:**
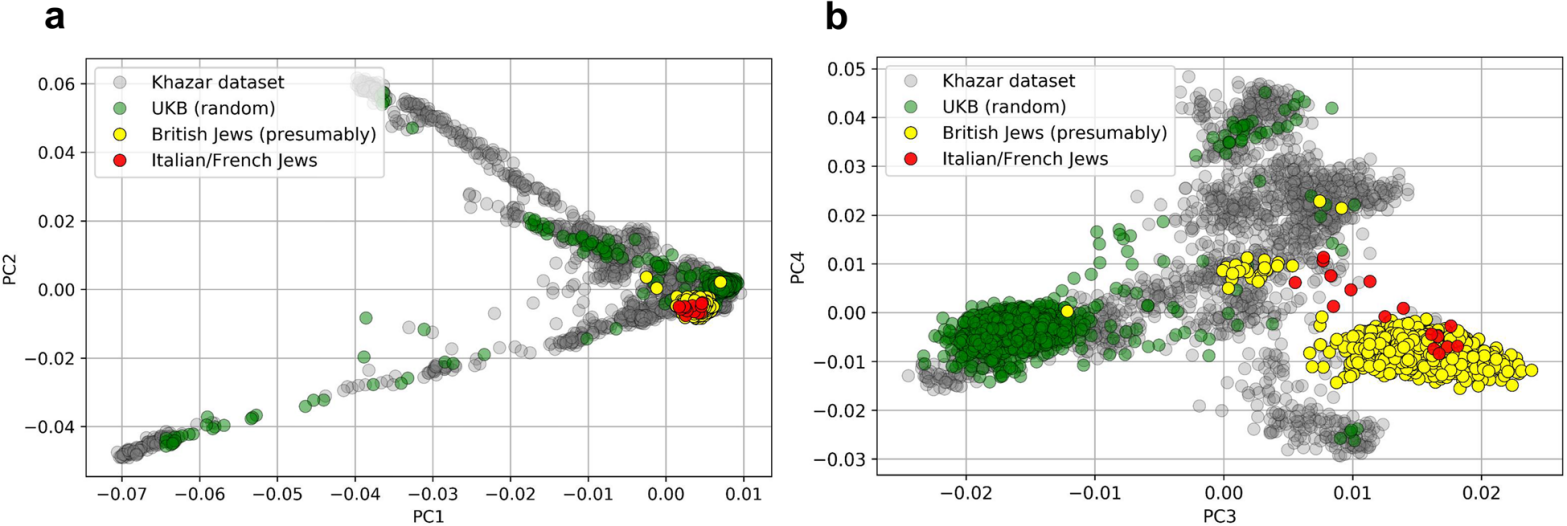
Principal components analysis of genetic variants of potential British Jews, the Khazar dataset, and a randomly selected subset of individuals from UK Biobank. PCA’s were calculated using the intersection of the genetic variants from the UK Biobank and the Khazar datasets. Forty principal components of the variance-standardized relationship matrix were extracted using PLINK v.1.9 [26] and the first four components were visualized: **a** first and second, **b** third and fourth principal components. The Italian and French Jews from the Khazar dataset are colored in red

In order to validate this hypothesis, we downloaded genotype data of an Ashkenazi Jewish individual and phased them using his parents’ genotypes. The trio’s data were downloaded from the Personal Genome Project [23]. We then searched for two-phased haplotypes of the son (in Chromosome 1) in the UKBB with a minimum target match length of 200 SNPs, corresponding to about 8 cM, using PBWT-Query [24]. A detailed description of the pipeline can be found in the “Methods” section. The first haplotype had 293 hits where 103 of them were in the subset of individuals with high *c* value. The second haplotype had 257, out of which 88 were in the subset of individuals with high *c* values. The fact that over 1/3 of hits were from this 0.4% population suggests that this cluster of high *c* values identifies probable British Jews (*p* value< 10^− 16^, chi-squared test). We further compared the potential Jews with European Jews in the Khazar dataset [25] using traditional Principal Component Analysis. The PCA results confirm that the cluster with high c values is indeed similar to the European Jews (Italian or French Jews) which further confirms our conjecture (see Fig. 4).

### Analysis of relative enrichment in neighborhood reveals regional patterns of preference for living closer to relatives

It is generally expected that an individual lives near to one’s extended family. Assuming relatives represent one’s extended family, we can study the social behavior of individuals. To quantify the preference of an individual towards living closer to their extended family, or local connectivity, we calculated the relative enrichment within the neighborhood (REIN). This can be defined as the ratio between the relative density in the neighborhood to the relative density across the entire UK. A REIN of one indicates no preference for local connectivity, and a larger REIN indicates a stronger preference. We confirmed that overall relative density is indeed enriched by almost 1.7 fold in the neighborhood of a 1 km radius of a person. This enrichment showed a sign of slow decay as the neighborhood radius became greater (Additional file 1: Table S6). REIN as a measure of local connectivity is quite noisy as indicated by its broad distributions (Additional file 1: Fig. S12). We chose to use relative enrichment within a 25 km radius (e25) as the REIN measure as it includes a greater number of individuals and thus is numerically more stable.

**Fig. 5:**
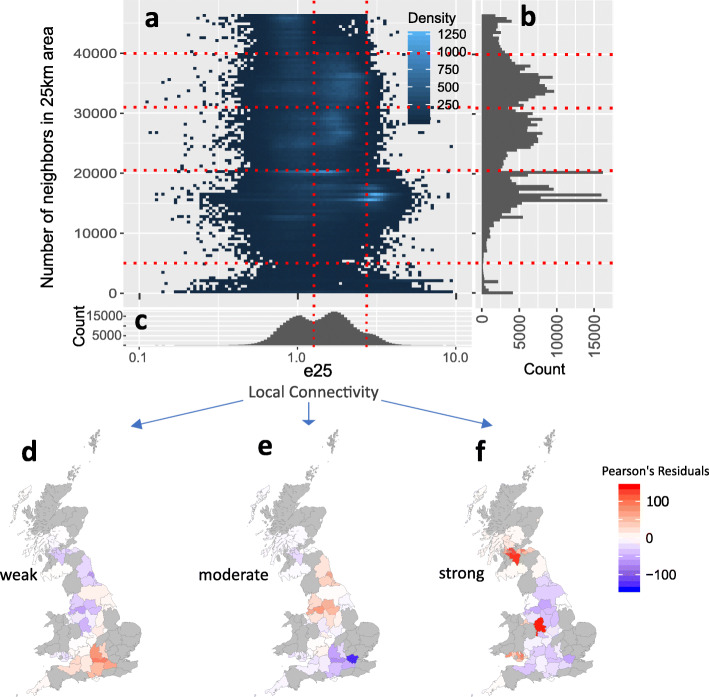
Preference of local connectivity as measured by relative enrichment in the neighborhood (REIN). **a** Preference of local connectivity (enrichment of relatives in a 25 km neighborhood, e25) is overall inversely correlated with population density (neighbor count). **b** Distribution of the number of neighbors in a 25 km area. **c** Distribution of the preference for local connectivity (enrichment of relatives in a 25 km radius). UK Biobank participants can be clustered into three groups using the local connectivity: people with preferences of weak, moderate, or strong local connectivity. The vertical bars (divisions of strong, moderate, and weak local connectivity) were determined by visual inspection. **d**–**f** Maps of regional enrichments of people with weak, moderate, and strong local connectivities. The enrichment of types of residents in each region was quantified by the Pearsons’ residuals: A positive residual (red) corresponds to enrichment while a negative residual (blue) indicates depletion. Counties with a low sampling rate (< 0.001) are colored in gray

Interestingly, local connectivity is inversely correlated with the 25 km neighbor count (Fig. 5 and Additional file 1: Fig. S13 and Fig. S14). Based on e25, we identify 3 clusters: people with weak, moderate, or strong local connectivity. Using the number of neighbors within 25 km, i.e., the density of UK Biobank participants in one’s neighborhood, as a proxy of the living environment, we divided the individuals as dwellers of five different regions based on the population density of their home location (Methods: the “Designating types of living environment by counting neighbors” section). For example, areas with more than 40,000 individuals are designated as high-density areas. Our analysis assumes sampling density is an approximation of the census density. For people in very high-density areas, there is a lack of local connectivity (weak local connectivity). For people living in high and moderate density areas (with the neighbor count in 21,000-40,000), there is a second subgroup of people with e25 = 1.7. For people with a neighbor count < 21,000, the relative enrichment in the neighborhood is very high, suggesting people living in less-populated areas tend to have higher enrichment of relatives within the neighborhood.

We further depict the regional differences of people with weak, moderate, and strong local connectivities (Fig. 5(d–f) and Additional file 1: Table S7). Interestingly, the counties that are with the highest enrichment for people with weak local connectivities are Greater London, Berkshire, and Oxfordshire, while the counties with the highest enrichment for people with strong local connectivity are Staffordshire, Greater Glasgow (South Lanarkshire, East Dunbartonshire, and North Lanarkshire) and Cardiff. Interesting, Greater Manchester, Merseyside, and Tyne and Wear are the areas with the highest enrichment of moderate local connectivity.

## Discussions

The main focus of the study is to provide a new data-driven descriptive analysis of the personal genealogical history of a large modern population using IBD sharing patterns. By analyzing the 3.5 billion IBD segments > 5 cM shared among half a million UK Biobank participants, our analysis offers a unique angle into the very recent demographic history of the UK. Unlike existing studies that focus on IBD segments between all individuals that reflect the history of the population [2, 10, 21], we focus on the personalized genealogical histories of individuals.

Based on approximately 14,000 IBD segments shared between a person and the rest of the UK Biobank participants, we discussed 3 categories of personalized genealogical descriptors: the genome coverage by IBD segments, the change of relative counts at different IBD lengths, and the relative enrichment within a neighborhood. Each of these genealogical descriptors reveals interesting details about personal genealogical history.

First, our analysis of genome coverage by IBD segments provides much higher resolution detail about the sharing of genetic information in a large modern population. We found that even with sampling the UK population at a rate of merely 0.7%, more than half of UK individuals have 80% of their genome potentially imputed using IBD segments shared with others. Our analysis offered intricate implications as to genome privacy. As the fast-growing business of direct-to-consumer (DTC) genomics, genome privacy has become of importance in both academia and the general public. Previously, concerns have been focused on the potential discrimination by employers and health insurance companies against carriers of certain mutations [27, 28]. Recently, a new concern [29] has been raised regarding law enforcement’s access to biobank scale genomic databases for solving criminal cases by connecting remote relatives genetically. Our study demonstrated that those two concerns are indeed two sides of the same coin as one’s genome can be largely reconstructed by the genomes of his/her genetic relatives traditionally thought as “unrelated.” Even sharing a low resolution of one’s genome, such as genotypes of 500 K non-clinically-associated SNVs used by a DTC genomics company, may grant access to a much more detailed genomic information at base-pair resolution. Therefore, public awareness of this issue of genome privacy is needed.

Second, our analysis of the variation of relative counts at different IBD lengths revealed individualized family expansion history. While some existing studies of population IBD patterns focused on clustering individuals based on the IBD network [10], we offered an individual-centered perspective. A unique benefit of our analysis is that instead of crawling the entire IBD network, such information can be collected efficiently via IBD queries such as PBWT-Query [24]. For example, we identified a minority group of individuals (likely British Jews) that went through drastic recent expansion. Of interest for potential future research is to derive theoretical frameworks for inferring more details of an individual’s family history.

Third, we also demonstrated that IBD information of individuals can be intersected with other information, such as geographic location, and offer new measurement of social behaviors. Using relatives as a proxy of one’s extended family, we showed that by studying the enrichment of relatives in one’s neighborhood, an individual’s preference towards staying with extended family (local connectivity) is revealed. Interestingly, drastic regional variations of preferences of local connectivity were shown. In Greater London, people with the weakest local connectivity are found, reflecting its cosmopolitan status. In Greater Manchester, people with moderate enrichment of local connectivity are found, indicating regional demographic movement in central England. In the major cities of Scotland and Wales, people with strong local connectivity are found. A limitation of our study is that UK Biobank is not necessarily a uniform random sampling of the UK population. The home addresses of participants are mostly clustered around recruitment centers (see Supplementary Fig. 1 in [17]). Therefore, validation of our results regarding the geographical locations on the rest of the UK regions may be needed.

In this work, we conducted a population genetics study at the population-scale, i.e., the study sample size approaches a significant proportion of the entire population. Traditional genetics studies are mostly at the sub-population scale, i.e., the samples under study were either a collection of “unrelated individuals” or familial members with traceable pedigrees. Population-scale data sets such as the UK Biobank offer the opportunity of studying emergent phenomena that are not possible at the sub-population scale.

This analysis offers an alternative definition of ancestry. Traditionally, the global ancestry of a person is typically labeled by one or several predefined continental-level groupings. Although local ancestries can add different labels across different genomic regions, each label is still of continental-level. Rather than resorting to these somewhat arbitrary labels, we offer an alternative way of describing a person’s ancestry label by using the geographic labels of one’s ancestors, e.g., their birth locations [30] or the patterns of shared IBD segments.

Based on our analysis, a person can use the geographic locations of his/her genetic relatives as a proxy to the locations of potential ancestors. For example, one UK person may be described as having 90% of genetic relatives living in Manchester, 9% in London, and 1% from Africa. By revealing regional genetic connections, our approach may open new research avenues for studying family-related social behaviors. This description can be enriched by adding temporal resolution by defining genetic relatives with different IBD length cutoffs. Of course, this is still imperfect. The number of ancestors of each individual grows quasi-exponentially with the number of generations in the near past; the genetic relatives identified by IBD segment analysis may be distributed broadly in a spectrum of generations. Also, the distribution of ancestors’ geographic locations may vary from generation to generation. Analyses of these patterns and theoretical modeling may be another exciting new research avenue.

## Conclusions

In this study, we investigate the individualized IBD sharing patterns of UK individuals using UK Biobank data. We found that since a substantial portion of the UK population is sampled, very dense IBD information is revealed by modern IBD detection methods. These IBD segments offer new insights into personalized genealogical history of individuals. We found a large fraction of an individual’s genome can be covered by shared IBD segments, which has implications for genotype imputation and privacy. Also, we found by looking at the relative counts of IBD segments shared at different lengths, we can reveal genealogical history of individuals, and thus enabling discovery of subpopulations with distinct genealogical patterns. Moreover, using people sharing IBD segments as a representative sample of one’s relatives, we can infer personal social behaviors such as preference favoring living closer to one’s extended families. In summary, our analysis revealed genetic makeup, personal genealogical history, and social behaviors at the population scale. While our current study is mostly descriptive in nature, we believe it opens possibilities for further studies of individual’s genetic connections in biobank data.

## Methods

### IBD segment calling using RaPID

The phased haplotypes in BGENv1.2 format from the UK Biobank release (version 2) were extracted and converted to VCF format. Phasing was performed by the UK Biobank team using SHAPEIT3, as described in [8]. RaPID v.1.7 was run for all 22 autosomal chromosomes of all phased haplotypes in the UKBB comprising 487,409 participants and 658,720 sites [8]. We did not use the imputed data as the imputed data may not lead to more accurate results. The data density would become crucial when searching for shorter IBD segments (e.g., 1 or 2 cM). The phased haplotypes from the UK Biobank V2 release were downloaded and converted to VCF files. The parameters for RaPID were calculated assuming a genotyping error rate of 0.25%. The number of runs was set to 10, the minimum number of passes to 2, and the window sizes to 3. The minimum target length was set to 5 cM (-r 10 -s 2 -w 3 -l 5). The genetic maps from deCODE [31] for hg38 were downloaded and lifted over to h19 using the liftOver tool [32]. The sites that did not maintain the genomic position order to their neighboring sites after conversion were discarded. The remaining sites were used to obtain the genetic locations of the available sites in the UKBB using linear interpolation.

### Runs of PBWTs over genetic distance (RaPID v.1.7)

We modified RaPID to allow direct use of genetic distances in PBWT [33]. The latest version of RaPID can take the minimum target length directly in cM and will return all detected segments greater than or equal to the given length without post-processing of data. The program holds a genetic mapping table for all the available sites and the PBWT has been modified to work directly with the genetic length instead of the number of sites. These changes were implemented in RaPID v.1.7.

We compared the results of the previous version of RaPID (v.1.2.3) and the new version (v.1.7) using the UK Biobank data. We found that the runtime has decreased from 12.78 days to 5.25 days, primarily due to the accurate control of window sizes.

### Benchmarking RaPID results versus KING

Related individuals from KING were compared to the individuals sharing IBD segments reported by RaPID. KING is a commonly used method for calling close relatives from global genotype similarity and thus provides an anchor for evaluating IBD segment calls. The relatedness data (up to third-degree relationship) from genotypes generated by KING were downloaded from the UK Biobank project [8]. In order to distinguish parent/offspring and full siblings pairs in the first-degree relationships, an IBS0 cutoff of 0.002 was selected. If the IBS0 value was greater than 0.002, then the pair was considered as full siblings.

### Benchmarking RaPID results versus GERMLINE, Refined IBD, and iLash

While it is possible to benchmark IBD segment detection methods using simulated data, it is almost impossible to capture all of the nuances in the real data. On the other hand, it is difficult to compare different methods within real data as there is often a lack of ground truth. We investigated the consistency among the IBD calls from different methods and then used the small subsets of MZ twin pairs for evaluation of detection power. The parameters for running GERMLINE and Refined IBD can be found in Additional file 1: Table S8.

We collected IBD results from four tools (GERMLINE, iLASH, Refined IBD, and RaPID) as four distinct sets of IBD segments: S_1_, S_2_, S_3_, and S_4_ respectively. All IBD segments that have been reported by at least one of the tools (in any of the result sets) were further investigated. To check whether an IBD1 in S_1_ is reported in S_2_, we searched in S_2_ for the same pair of individuals and checked if there is any reported IBD segment that overlaps at least 50% or more of the reported segment in S_1_. The percentage of IBD segments in each set that has been covered by other tools have been reported in the Additional file 1: Table S1. This is not a systematic evaluation of the pros and cons of individual methods. Still, consistent IBD segment calls indicate a degree of confidence. Inconsistent calls among methods would warrant some additional investigation.

### Estimation of detection power and accuracy

To estimate the detection power of different methods, 179 pairs of MZ twins (reported by KING) were considered. We expected that the reported IBD segments would cover the entire chromosomes between any two MZ twins. Due to the stochastic nature of current phasing methods, there are phasing errors even between MZ twins. The detection power for each MZ twin pair was defined as the percentage of the genome (all 22 chromosomes) that has been covered by reported IBD segments. The average detection power values between all MZ twins among British, non-British, and all pairs were reported. The field 21,000 from the UK Biobank data was used to determine the ethnic backgrounds of the MZ twins. For Refined IBD, we ran the program only once. Running Refined IBD with different seeds and merging results may increase the detection power but at the same time, it will require more computational resources. For GERMLINE, we used the tag -h_extend which is designed for well-phased data. Therefore, it will be less tolerable to phasing errors but no false positives are introduced as a result of tolerating phasing errors.

Overall, there are low chances that a false positive segment over 5 cM is called by any of these methods. To investigate the accuracy of the reported segment implicitly, we extracted 5674 parent/offsprings (identified by KING) with the self-reported British background. Assuming there is no inbreeding, reported IBD segments between parent and offspring should not overlap. Please note that multiple IBD segments in each chromosome may be reported between an offspring and its parent due to recombinations or phasing error. We collected all 5674 parents/offspring pairs identified by KING. We searched for potential false positives, i.e., the overlapping IBD segments between parent/offspring pairs. An IBD segment is considered to be false positive if 50% of the segment is covered by another IBD segment between the same pair of parents/offspring. We find < 0.01% potential false positives in any of these methods. If we defined the accuracy as percentage correctly identified segments: accuracy = (#IBD - #false positives)/(#IBD), then the accuracy values for RaPID, GERMLINE, iLash, and Refined IBD using parent/offspring pairs would be 99.8740%, 99.9803%, 99.9217%, and 99.9126%, respectively. Manual inspection of false positives also revealed that most cases are also likely due to runs-of-homozygous segments.

### Trio consistency

The consistency of reported IBD segments among trios were investigated similar to [15]. The consistency check is based on the observation that if a child has an IBD segment with another individual, then at least one of the parents should also share the IBD segment with the other individual. One hundred thirty-three parent/child trios were extracted from 200,000 individuals in the UK Biobank. We then investigated the percentage of child-other IBD segments that were covered by at least one of the parents of the child (we call parent-coverage overlap rate). While Durand et al. [15] used a minimal overlap cutoff to define a binary overlap/nonoverlap label for each segment, we defined a non-binary fraction of child-other segments covered by parent-other pair, to allow a single number to describe the trio consistency.

### Assignment of the geographic area

Ordinance coordinates in the form of (east, north) were retrieved from the UKBB fields 22702 and 22704. The coordinates were converted to longitudes and latitudes using the Python library *convertbng* (https://pypi.org/project/convertbng/). The Python library *reverse_geocoder* (https://pypi.org/project/reverse_geocoder/) was then used to find the nearest town/city using the GPS coordinates. For each coordinate, *reverse_geocoder* returns only the corresponding subdivision, which is finer than counties. The subdivisions were then translated into the corresponding counties.

### Relative count adjustment by the regional sampling rate

The population size for each county was extracted [34]. For each county *i*, the sampling rate *S*_*i*_ was defined as the number of participants divided by the population size. For each individual, the number of relatives in each county (*C*_*i*_) was calculated (using the home locations). The normalized number of cousins for each individual was then calculated using the following formula: $C=r{\sum}_{\mathrm{i}=1}^N{C}_i{S}_i^{-1}$, where *N* denotes the total number of counties. The quantity ${\sum}_{\mathrm{i}=1}^N{C}_i{S}_i^{-1}$ denotes the total number of (sampled and unsampled) cousins the person has, and the quantity *r* denotes the average sampling rate for all counties, i.e., 0.7%.

### Searching for query haplotype from the Personal Genome Project in UKBB

The trio data of an Ashkenazi Jewish family (huAA53E0 son and his parents hu8E87A9, hu6E4515) were downloaded [35]. The sites containing SNPs were extracted from the trio data. Each site containing a missing value was discarded. The genotype data for the son were then phased using parent data and simplified by filtering out all-het sites. The overlapping sites with the UKBB (chromosome 1) were selected which resulted in 6629 sites. The 6629 sites of chromosome 1 for all the individuals from UKBB were extracted using vcftools (v0.1.15) [36]. PBWT-Query [24] was used to search for the query haplotypes with a minimum target length of 200 SNPs (-L 200).

### Genetic comparison of potential British Jewry with Khazar dataset

The full Khazar dataset [25] was downloaded and combined with 1719 potential Jews and another 1719 randomly selected from the UK Biobank. The combined dataset contains 46,215 sites across all autosomal chromosomes. 427 sites from the Khazar dataset were flipped using Plink (-flip) due to strand inconsistency. Plink was used to compute PCA for the first 40 components and the first 4 components were presented.

### Design of genetic genealogical descriptors

The following categories of genetic genealogical descriptors were defined to capture extensive information from the relative cohorts of an individual: (1) percentage of the genome covered by IBD segments from relatives, (2) decay of relative counts as the length of IBD segments increase, and (3) enrichment of relatives in one’s neighborhood.

### Enrichment of counts in tables

For describing the enrichment of counts of individuals in a contingency table, e.g., e25 vs city_size or c vs area, we used the Pearson’s residual: ((observed − expected)/sqrt (expected)).

### Percentage of the genome covered by IBD segments

To investigate the correlation between the available number of individuals from a population and the percentage of the genome covered, 10 subsets {S_1_, S_2_,.., S_10_} of the UK Biobank were extracted. S_1_ contains all available 480,518 individuals. S_i + 1_ was generated by randomly selecting half of the individuals from S_i_. For each subset, the average genome coverage was computed as follows: each chromosome was divided into bins of 1 Mbps. For each individual, the number of shared IBD segments overlapping with each bin was calculated. Then, the number of bins overlapping with at least 1, 5, and 10 IBD segments were calculated and divided by the total number of bins. Finally, the average of the genome coverage among all available individuals was computed.

### Collecting IBD segment counts at different lengths

For each individual, counts of relatives sharing IBD segments at different lengths are informative to personal genealogical history. We chose the counts of IBD segments in [5, 10), and [10, 3400). The total sum of IBD segments between any two pairs of individuals sharing an IBD segment (relative) from all autosomal chromosomes in the UKBB was calculated. Subsequently, the number of relatives of each individual for two bins (5–10 cM and ≥ 10 cM) was computed.

### Enrichment of relatives in one’s neighborhood

The UK Biobank home location fields 22,702_0_0 (east coordinate) and 22,704_0_0 (north coordinate) were used to compute the distance between any two individuals. The number of neighbors and relatives within a neighborhood of 1, 5, 10, and 25 km were calculated as follows: for each individual and the given radius *r* (e.g., 1 km), all UK participants within a distance *r* from both query’s east and north coordinates were extracted. Subsequently, the Euclidean distances between the query individual and the extracted individuals were calculated.

### Designating types of living environment by counting neighbors

By plotting the histogram of the number of neighbors within 25 km (Fig. 4b), we divided the individuals as dwellers of regions with very high, high, moderate, low, and very low density, as people with the count of neighbors included in UK Biobank > 40,000, 31,000–40,000, 21,000–31,000, 5000–21,000, and < 5000, respectively.

## Supplementary Information

**Additional file 1: Figure S1.** The probability distributions of the sum of genetic lengths shared among pairs of individuals in five types of relatedness using detected IBD segments by RaPID. **Figure S2.** Number of IBD calls for different IBD detection tools using chromosome 22 of 200K individuals from UK Biobank. **Figure S3.** An example of IBD segments over chromosome 12 called by different methods using a twin pair. **Figure S4.** Average detection power of different methods for twins on all autosomes. **Figure S5.** Ethnicity by ethnicity kinship matrix using the self-reported ethnic backgrounds in UK Biobank and the sum of detected IBD segments by RaPID in all autosomes. **Figure S6.** Relative count of British individuals**. Figure S7.** Cross-region average relative count. Numbers are normalized by total potential pairs and the total length of the chromosomes. **Figure S8.** Percentage of the genome covered by IBD segments from others in UK Biobank by ethnicity. **Figure S9.** Average percentage coverage of chromosome 1 by IBD segments after filtering out detected IBD segments. **Figure S10.** Genome coverage of individuals by IBD segments in the UK Biobank data for chromosome 1. **Figure S11.** Number of relatives sharing 5-10 cM vs. sharing 10 cM IBD segments for individuals in UK Biobank after filtering RaPID results. **Figure S12.** The distribution of REIN of all UK Biobank participants within 1, 5, 10, and 25 km radius. **Figure S13.** Preference of local connectivity as measured by relative enrichment in the neighborhood (REIN) after filtering RaPID results. **Figure S14.** Correlation between the preference of local connectivity (enrichment of relatives in a 25 km neighborhood, e25) and population density (neighbor count). **Table S1.** IBD results of Germline, iLash, Refined IBD, and RaPID (rows) covered by other tools (columns). **Table S2.** Average detection power of different methods. **Table S3.** Accuracy of called IBD segments using MZ twin pairs. **Table S4.** Average mismatch rate and parent coverage of IBDs in trios using different methods. Average parent coverage denotes the proportion of the IBD segments from child-other that were overlapping by parent-other segment. **Table S5.** Parent coverage of IBDs in trios using different methods where parents are not used for phasing the genotype data. **Table S6.** Relative enrichment in different neighborhoods decays with increasing radius. **Table S7.** Enrichment of people with different local connectivities in UK areas, as indicated by Pearson’s residuals. **Table S8.** Parameters and command lines for benchmarking different IBD detection tools.

## Data Availability

The Khazar dataset was downloaded from https://evolbio.ut.ee/khazar/. The UK Biobank data are available from the UK Biobank study but restrictions apply to the availability of these data, which were used under license for the current study, and so are not publicly available. All other data generated in this study are included in this published article and its supplementary information files.
