# Supplementary material for: Personalized genealogical history of UK individuals inferred from biobank-scale IBD segments
Source: BMC Biol. 2021 Feb 16;19:32. doi: 10.1186/s12915-021-00964-y (PMC7888130; doi:10.1186/s12915-021-00964-y)
Supplement: Supplementary file 1 — Additional file 1: Figure S1. The probability distributions of the sum of genetic lengths shared among pairs of individuals in five types of relatedness using detected IBD segments by RaPID. Figure S2. Number of IBD calls for different IBD detection tools using chromosome 22 of 200K individuals from UK Biobank. Figure S3. An example of IBD segments over chromosome 12 called by different methods using a twin pair. Figure S4. Average detection power of different methods for twins on all autosomes. Figure S5. Ethnicity by ethnicity kinship matrix using the self-reported ethnic backgrounds in UK Biobank and the sum of detected IBD segments by RaPID in all autosomes. Figure S6. Relative count of British individuals. Figure S7. Cross-region average relative count. Numbers are normalized by total potential pairs and the total length of the chromosomes. Figure S8. Percentage of the genome covered by IBD segments from others in UK Biobank by ethnicity. Figure S9. Average percentage coverage of chromosome 1 by IBD segments after filtering out detected IBD segments. Figure S10. Genome coverage of individuals by IBD segments in the UK Biobank data for chromosome 1. Figure S11. Number of relatives sharing 5-10 cM vs. sharing 10 cM IBD segments for individuals in UK Biobank after filtering RaPID results. Figure S12. The distribution of REIN of all UK Biobank participants within 1, 5, 10, and 25 km radius. Figure S13. Preference of local connectivity as measured by relative enrichment in the neighborhood (REIN) after filtering RaPID results. Figure S14. Correlation between the preference of local connectivity (enrichment of relatives in a 25 km neighborhood, e25) and population density (neighbor count). Table S1. IBD results of Germline, iLash, Refined IBD, and RaPID (rows) covered by other tools (columns). Table S2. Average detection power of different methods. Table S3. Accuracy of called IBD segments using MZ twin pairs. Table S4. Average mismatch rate and parent coverage of IB [file 12915_2021_964_MOESM1_ESM.pdf]

Additional file 1

Personalized genealogical history of UK individuals inferred from biobank-scale IBD segments

Ardalan Naseri[1], Kecong Tang[2], Xin Geng[1], Junjie Shi[1], Jing Zhang[1], Pramesh Shakya[2], Xiaoming Liu[3], Shaojie Zhang[2,*], Degui Zhi[1,4*]

[1] School of Biomedical Informatics, The University of Texas Health Science Center at Houston, Houston, Texas, 77030, USA
[2] Department of Computer Science, University of Central Florida, Orlando, Florida, 32816, USA
[3] USF Genomics, College of Public Health, University of South Florida, Tampa, Florida, 33612, USA
[4] Center for Precision Health, School of Biomedical Informatics, School of Public Health, The University of Texas Health Science Center at Houston, Houston, Texas, 77030, USA

*Correspondence: shzhang@cs.ucf.edu or degui.zhi@uth.tmc.edu

Supplementary Figures

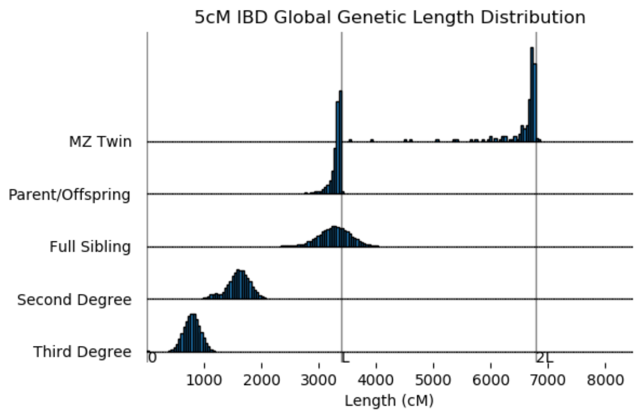

Fig. S1: The probability distributions of the sum of genetic lengths shared among pairs of individuals in five types of relatedness using detected IBD segments by RaPID with a target length of 5 cM and above using all autosomal chromosomes. The degree of relatedness was determined by KING. The visible outliers in the MZ Twin track are non-British people from the UKBB which are of lower genotype and phasing quality and thus the full-length calls are difficult (also supported by Supplementary Figure S4).

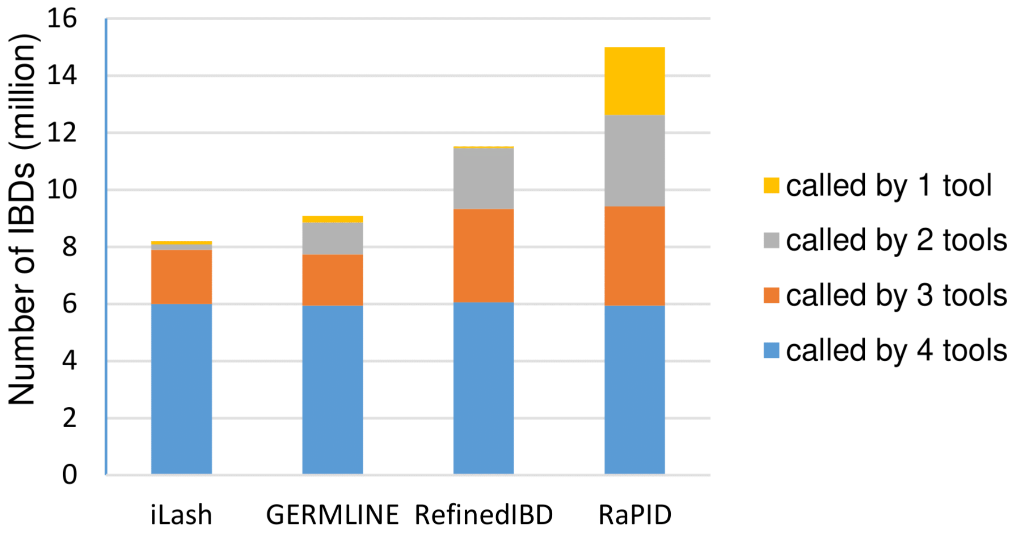

Fig. S2: Number of IBD calls for different IBD detection tools using chromosome 22 of 200K individuals from UK Biobank. IBD calls for each tool are stratified by the total number of tools that identified the segments.

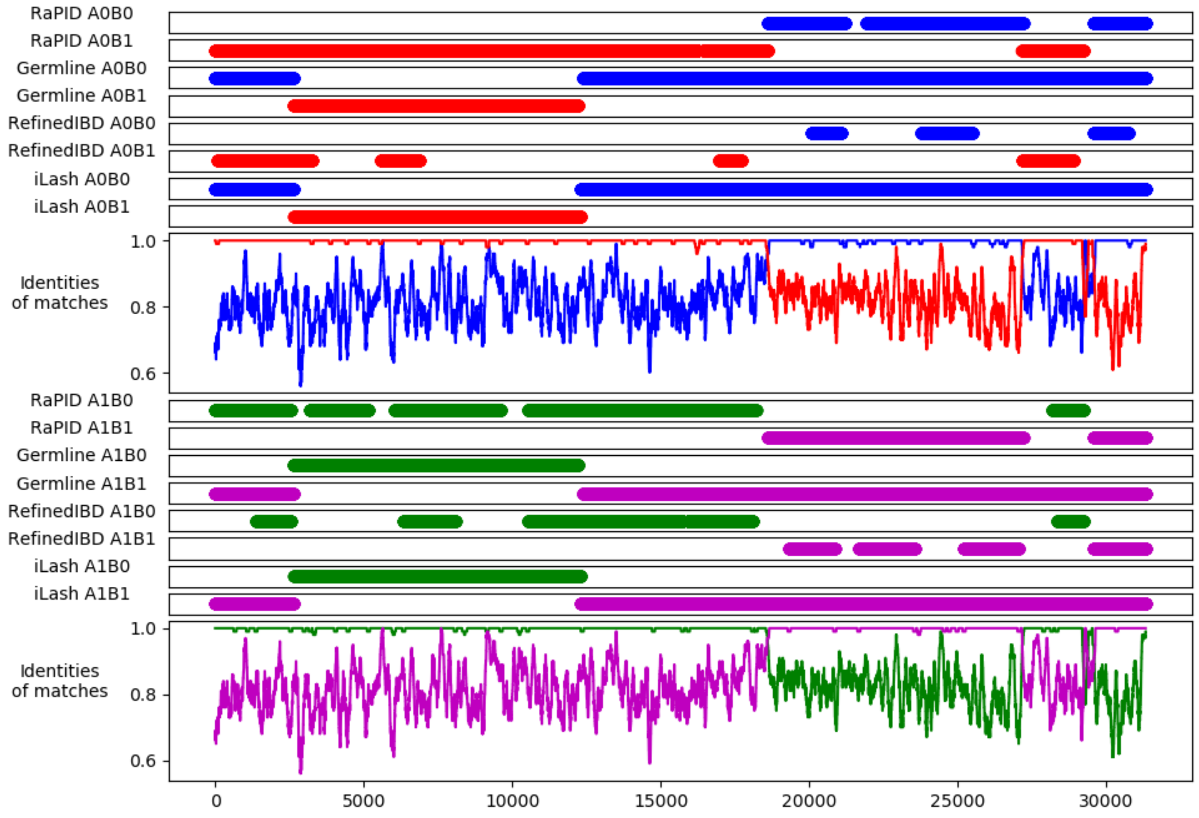

Fig. S3: An example of IBD segments over chromosome 12 called by different methods using a twin pair. The identities of matches over the moving averages of windows with 100 SNPs were computed using four different combinations of haplotypes: A0B0, A0B1, A1B0, and A1B1. A0B0 matches are represented in blue, A0B1 in red, A1B0 in green, and A1B1 in purple. IBD results for each tool have been depicted on the top tracks. The x-axis denotes the site index of chromosome 12.

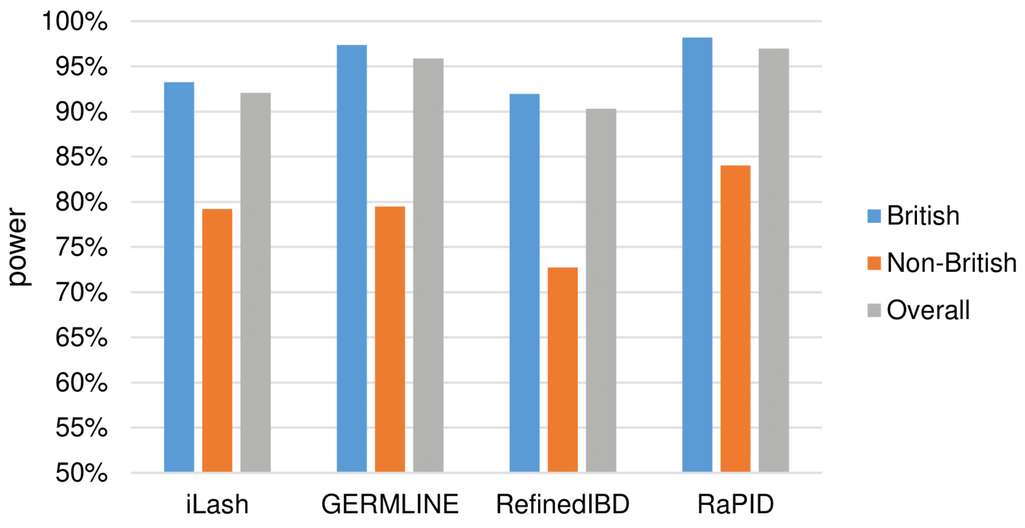

Fig. S4: Average detection power of different methods for twins on all autosomes.

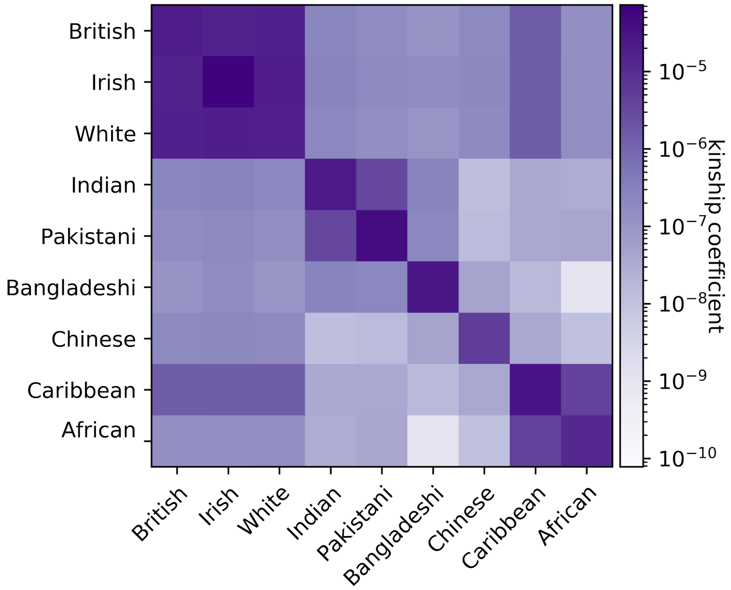

Fig. S5: Ethnicity by ethnicity kinship matrix using the self-reported ethnic backgrounds in UK Biobank and the sum of detected IBD segments by RaPID in all autosomes. White refers to any other background excluding British or Irish. Intrapopulation kinship values are higher than interpopulation kinship values, as expected. The kinship value between British and Irish is high, however, the Irish population has a distinguishable intrapopulation kinship. The closest population to Indian is Pakistani according to kinship values. The African population has the highest kinship with the Caribbean among the other ethnic groups as anticipated.

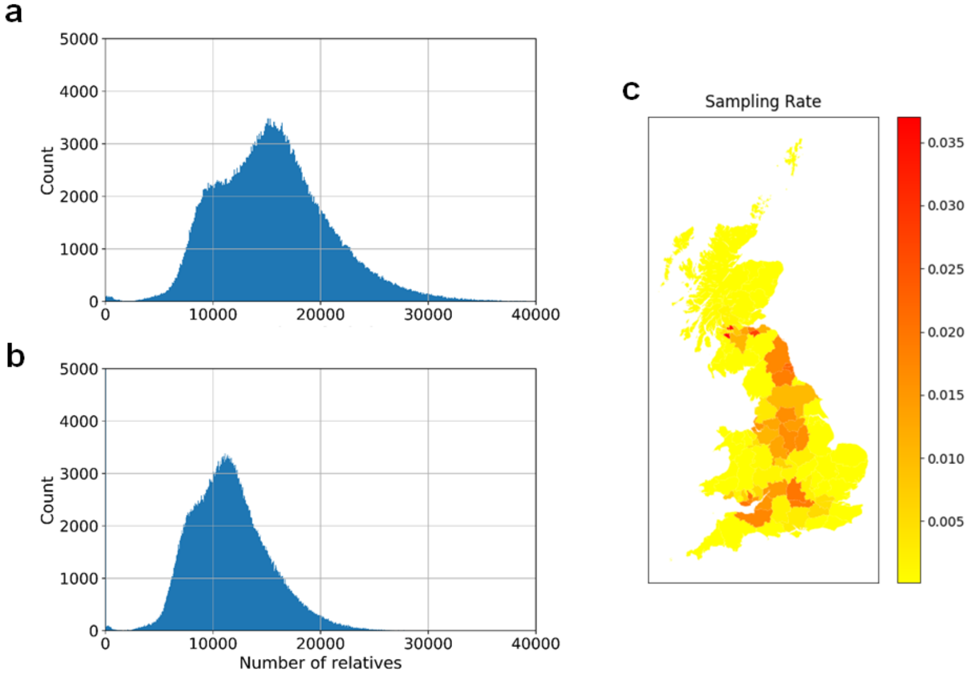

Fig. S6: Relative count of British individuals. **(a)** Unadjusted. **(b)** Adjusted by regional sampling rates. **(c)** The sampling rates of the UK counties using the home location of UK Biobank participants.

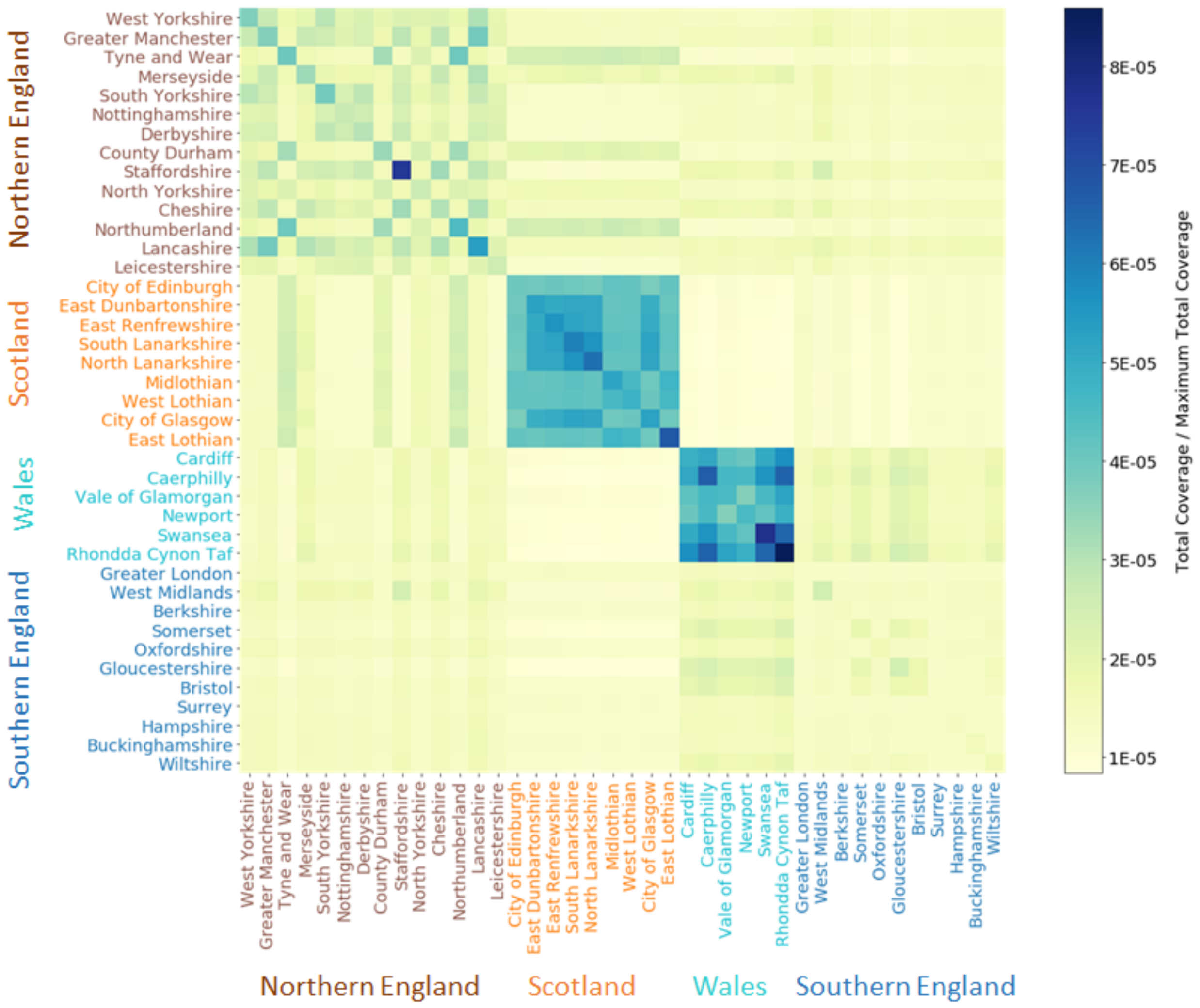

Fig. S7: Cross-region average relative count. Numbers are normalized by total potential pairs and the total length of the chromosomes.

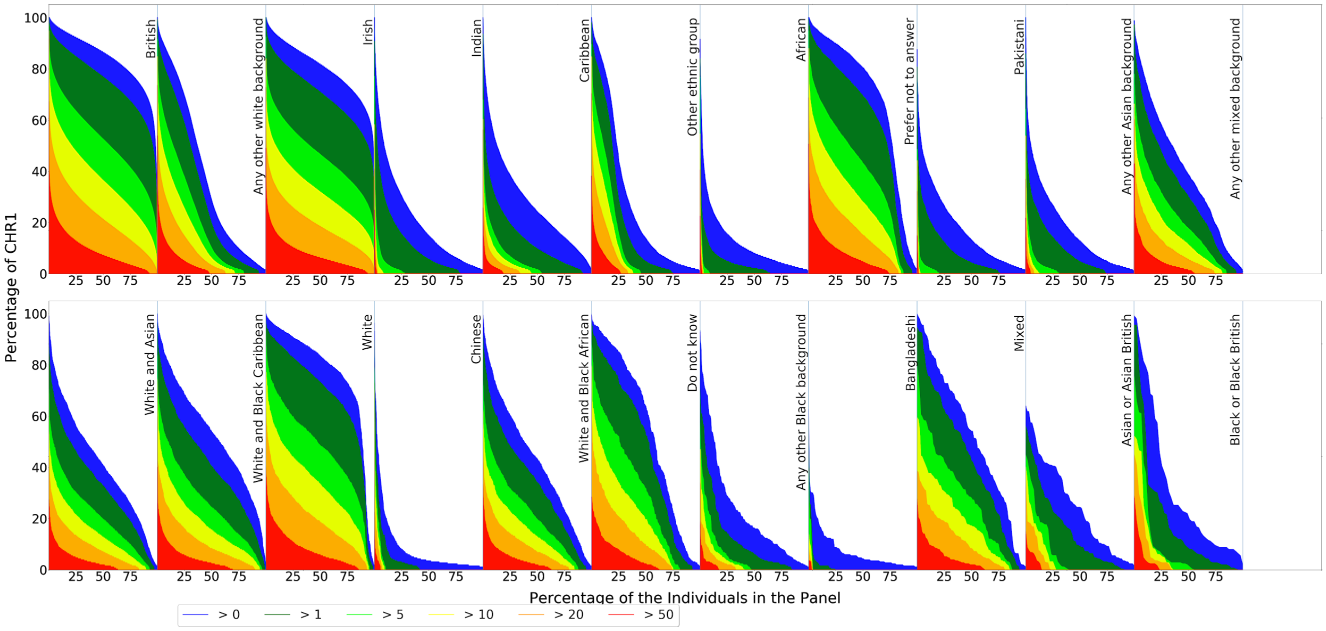

Fig. S8: Percentage of the genome covered by IBD segments from others in UK Biobank by ethnicity.

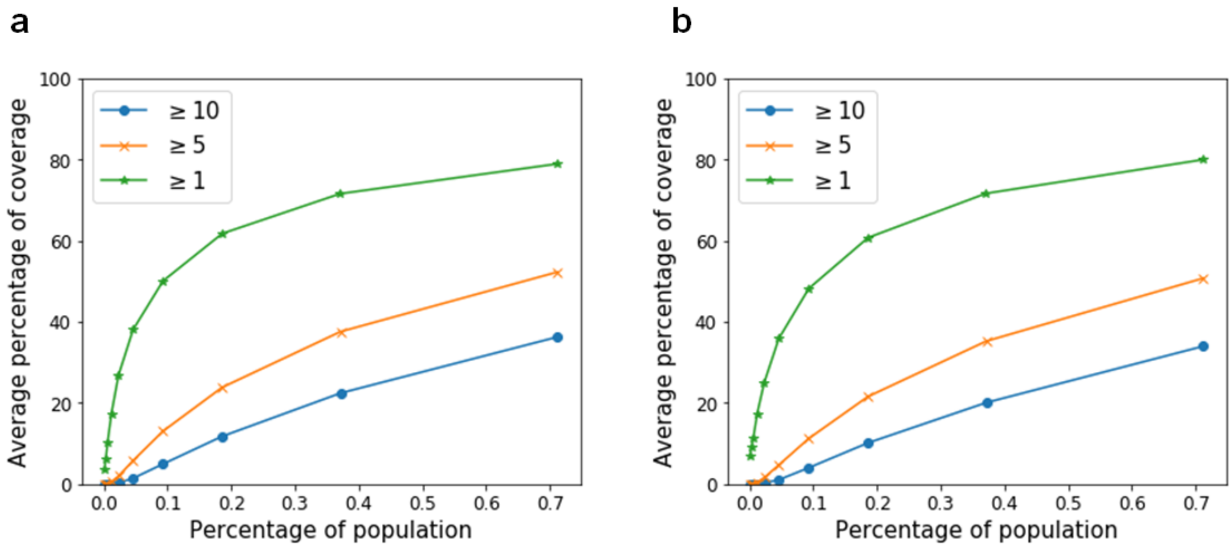

Fig. S9: **(a)** Average percentage coverage of chromosome 1 by IBD segments after filtering out detected IBD segments with a high mismatch rate. The Percentage of the population is calculated by considering the available proportion of genotyped individuals from the UK population. Overall ~52% (225010 out of 430189) of the British have over 85% of their genome and ~72% (308315 out of 430189) have over 80%, covered by at least 1 IBD segment. For coverage of 10 IBD segments, 46% of British individuals (199793 out of 430189) have over 40% covered by at least 10 segments. **(b)** Average percentage of coverage of first 50 Mbps of chromosome 1 using RaPID results that were called by iLASH or RefinedIBD.

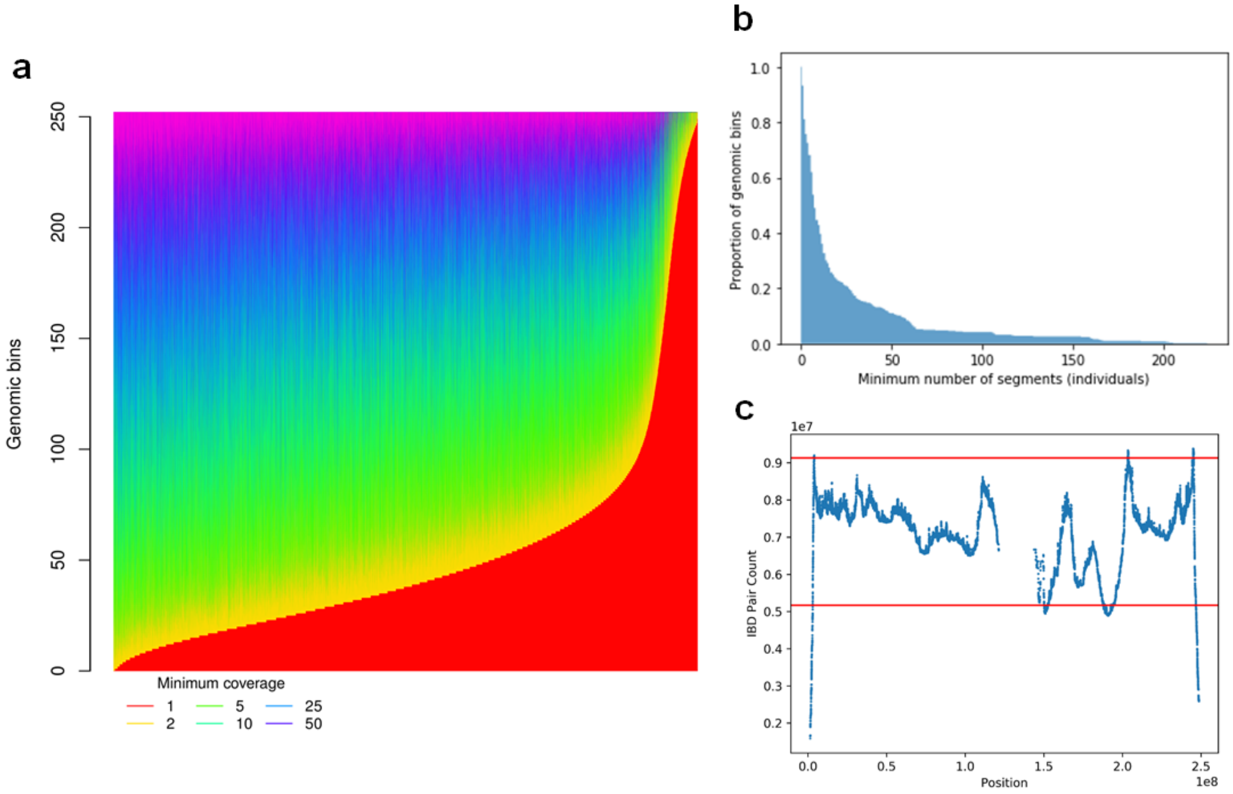

Fig. S10: Genome coverage of individuals by IBD segments (>=5 cM) in the UK Biobank data for chromosome 1. **(a)** Number of genomic bins covered in chromosome 1 by 1, 2, 5, 10, 25, and 50 IBD segments (or more). The x-axis represents individuals sorted by the number of genomic bins covered by 1 segment. **(b)** The proportion of genomic bins in chromosome 1 of one target individual covered by shared IBD segments with other individuals in the UK Biobank. **(c)** Genome coverage by IBD segments across chromosome 1 among all UK Biobank participants. Red lines mark the +/-4 standard deviations of the mean of the chromosomal coverage. Position denotes the genomic location in chromosome 1.

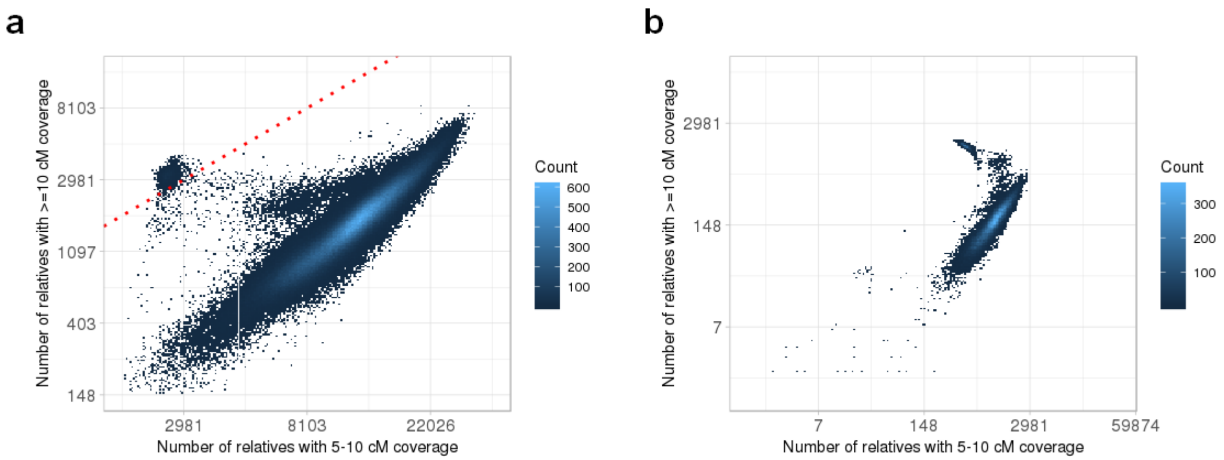

Fig. S11: Two clusters of individuals are separable sharing 5-10 cM and sharing 10 cM IBD segments even after filtering RaPID results **(a)** and the intersection of IBD calls between RaPID and RefinedIBD, iLASH or GERMLINE using a subset panel **(b)**. Since it was not feasible to run all tools using the entire UKBB, 50k UK individuals and 1719 putative British Jews (detected by RaPID previously) were considered. RaPID, RefinedIBD, iLASH and GERMLINE were run on the subset panel (50k + 1719). RaPID results that were not called by any other tool (RefinedIBD, GERMLINE, or iLASH) were filtered out.

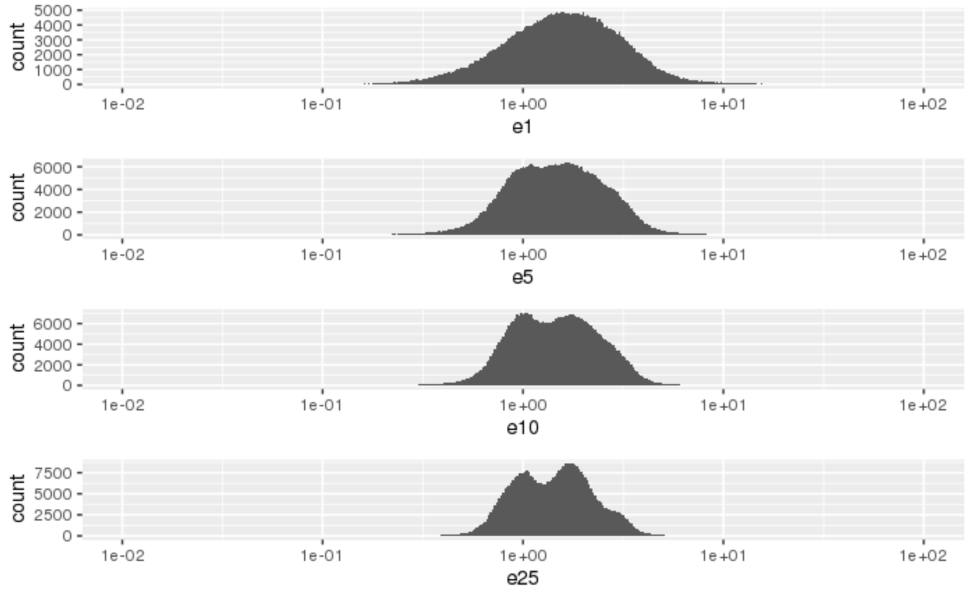

Fig. S12: The distribution of REIN of all UK Biobank participants within 1, 5, 10, and 25 km radius. eX denotes the relative enrichment within an X km radius.

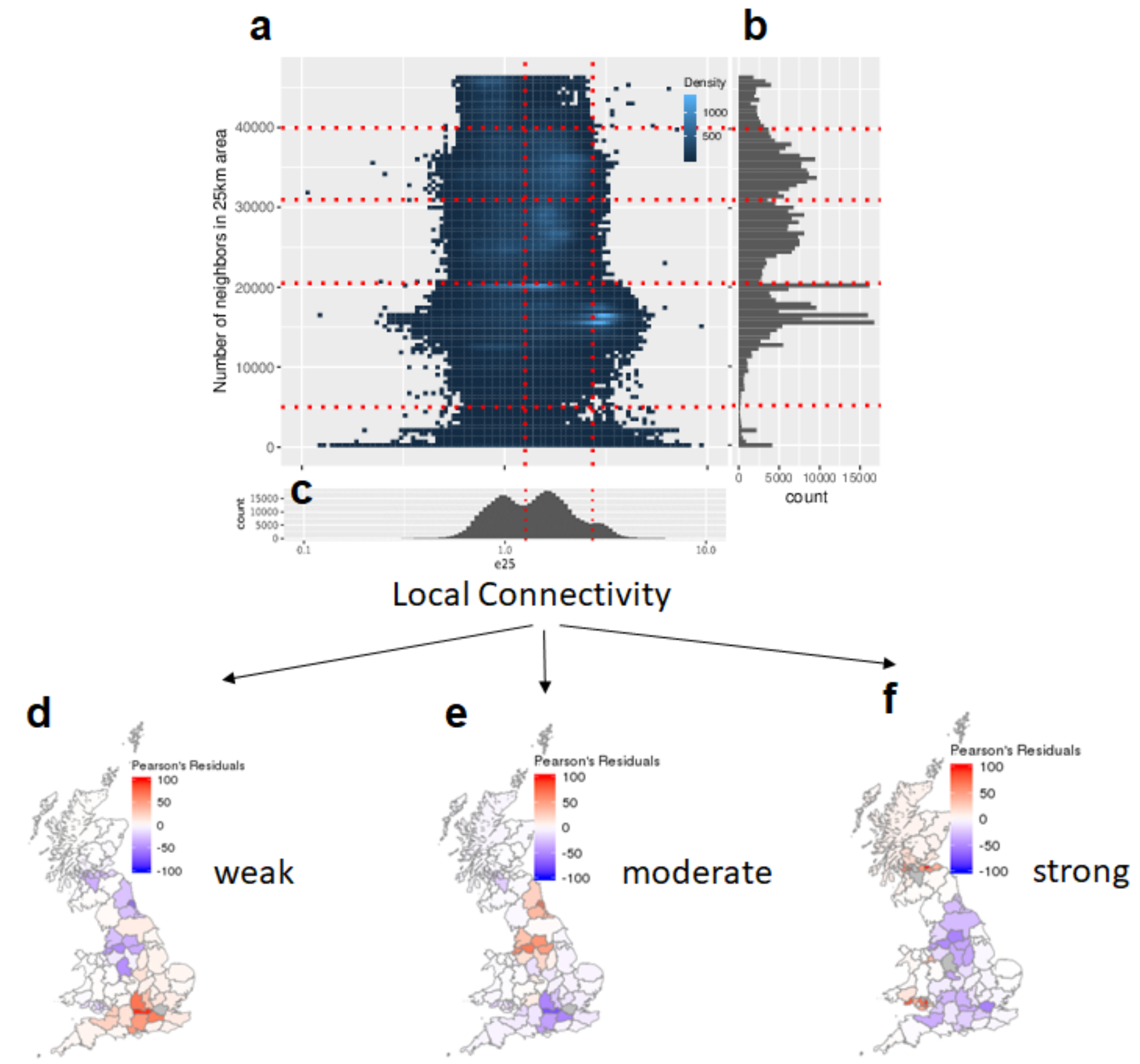

Fig. S13: Preference of local connectivity as measured by relative enrichment in the neighborhood (REIN) after filtering RaPID results. **(a)** Preference of local connectivity (enrichment of relatives in a 25 km neighborhood, e25) vs. population density (neighbor count). **(b)** The type of neighborhood of an individual can be captured by the number of neighbors in a 25 km radius. **(c)** Distribution of cousin enrichment in a 25 km neighborhood. **(d)(e)(f)** Maps of regional enrichment of people of weak, moderate, and strong local connectivities.

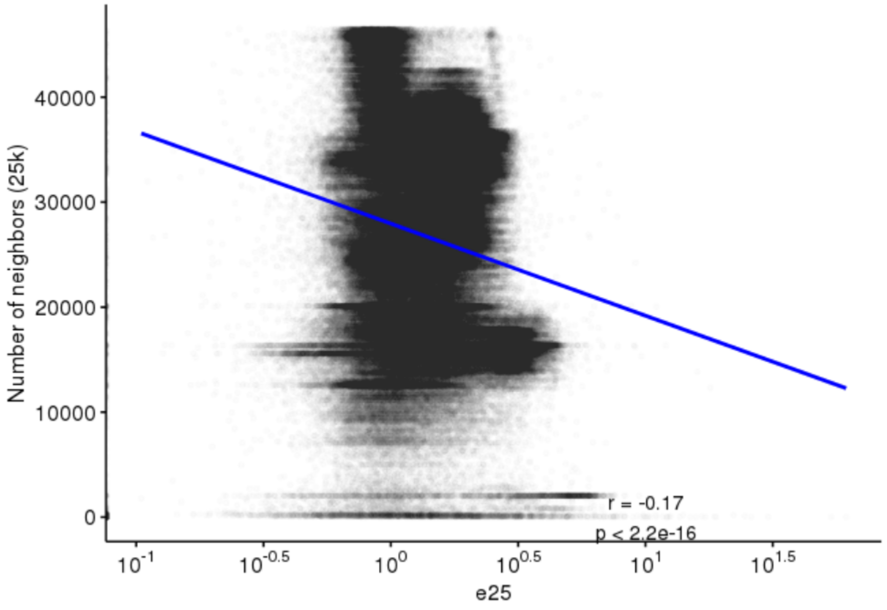

Fig. S14: Correlation between the preference of local connectivity (enrichment of relatives in a 25 km neighborhood, e25) and population density (neighbor count).

Supplementary Tables

Table S1: IBD results of Germline, iLash, RefinedIBD, and RaPID (rows) covered by other tools (columns).

**Chr.22**	**Covered by GERMLINE**	**Covered by iLASH**	**Covered by RefinedIBD**	**Covered by RaPID**
**GERMLINE**		68.53%	82.51%	95.76%
**iLASH**	77.27%		93.61%	97.24%
**RefinedIBD**	66.60%	67.13%		99.45%
**RaPID**	58.49%	52.75%	75.57%	

Table S2: Average detection power of different methods.

	British (n=164)	Non-British (n=15)	Overall (n=179)
**RaPID**	98.17%	84.03%	96.98%
**GERMLINE**	97.36%	79.48%	95.86%
**RefinedIBD**	86.02%	63.97%	84.18%
**iLash**	93.23%	79.20%	92.06%

Table S3: Accuracy of called IBD segments using MZ twin pairs. Results are based on the 200,000 subset panel of UK Biobank participants using Chr 22.

Method	Accuracy
**Germline**	99.93%
**RaPID**	99.91%
**RefinedIBD**	99.95%
**iLash**	99.96%

Table S4: Average mismatch rate and parent coverage of IBDs in trios using different methods. Average parent coverage denotes the proportion of the IBD segments from child-other that were overlapping by parent-other segment. Results are based on the 200,000 subset panel of UK Biobank participants using chromosome 22. Mismatch rate is the percentage of mismatching SNPs between the two haplotypes that are called IBD. RaPID All denotes all IBD segments detected by RaPID. RaPID Shared denotes IBD segments that overlap with any other tool. The mismatch rate for unique RaPID results for the core segment was 0.19%.

**Methods**		**Mismatch rate**	**# Child-other pairs**	**Average parent coverage**
**Germline**		0.06%	11197	98.27%
**iLash**		0.03%	10311	95.02%
**RefinedIBD**		0.00%	14413	93.63%
**RaPID**	All	0.41% (0.08%*)	20755	98.51%
	Shared	0.38% (0.05%*)	15895	95.92%

*core segment (90% middle part of the reported segment).

Table S5: Parent coverage of IBDs in trios of different methods after rephasing where parents are not used for phasing the genotype data. The genotype data of both parents in trios were removed and the remaining genotype data in UK Biobank were phased using SHAPEIT3. For the sake of simplicity, the parent's haplotypes were copied from UK Biobank haplotype release data.

**Methods**		**# Child-other pairs**	**Average parent coverage**
**Germline**		10954	98.42%
**iLash**		9181	94.86%
**RefinedIBD**		17984	93.93%
**RaPID**	All	20653	98.03%
	Shared	17025	98.72%

Table S6: Relative enrichment in different neighborhoods decays with increasing radius.

**radius (km)**	**1**	**5**	**10**	**25**
**average #neighbors**	295	2851	8438	26528
**average relative enrichment**	1.703	1.650	1.613	1.588

Table S7: Enrichment of people with different local connectivities in UK areas, as indicated by Pearson's residuals.

Area	Weak	Moderate	Strong
Aberdeen City	-0.1	-3.4	8.8
Aberdeenshire	1.6	-2.5	2.5
Angus	0.8	-2	3.3
Argyll & Bute	1.1	-3.3	5.8
Avon	27.5	-16.1	-22.8
Bedfordshire	5.8	-4.5	-1.9
Berkshire	92.7	-70.3	-36.5
Blaenau Gwent	-1.7	-0.8	6
Bridgend	-1.2	-1.2	5.7
Buckinghamshire	30.5	-23.3	-11.5
Caerphilly	-23.1	-15.5	92.1
Cambridgeshire	6.2	-4.9	-1.9
Cardiff	-20.4	-12.5	78.6
Carmarthenshire	-1.9	-3.4	12.8
Ceredigion	2.6	-3	1.5
Cheshire	-13.8	19.1	-16.2
Clackmannanshire	-1.1	-1.4	5.9
Conwy	1.8	-2.2	1.4
Cornwall and Isles of Scilly	5	-5.7	2.8
Cumbria	4.6	-4.5	0.9
Denbighshire	0.6	-0.9	1
Derbyshire	-9.4	23	-36.2
Devon	7.2	-6.9	0.7
Dorset	4.3	-4.4	1.1
Dumfries & Galloway	1.5	-3.1	4.4
Dundee City	0.5	-3.1	6.6
Durham	-25.6	33.2	-24.6
East Ayrshire	-2.1	-0.6	6.4
East Dunbartonshire	-30.5	-21.7	124.6
East Lothian	-15.2	0.3	34.2
East Renfrewshire	-30.3	-21.3	123.3

East Sussex	6.2	-5.1	-1.6
Edinburgh, City of	-34.9	-4.3	91.1
Eilean Siar	1.4	-2.2	2.5
Essex	7.8	-6.1	-2.7
Falkirk	-3.1	-2	12.3
Fife	-4.3	-0.5	11
Flintshire	-3.1	3.8	-2.3
Glasgow City	-21	-14.9	85.7
Gloucestershire	4.1	7.8	-29.2
Greater London	163	-127.8	-54.4
Greater Manchester	-57.3	72.2	-49.9
Gwynedd	2.2	-3.3	3.2
Hampshire	49.6	-37.6	-19.7
Hereford and Worcester	1.2	2	-7.9
Hertfordshire	12.2	-9.4	-4.5
Highland	2.6	-4	4.1
Humberside	5.7	-5	-0.7
Inverclyde	-2.5	-1.5	9.5
Isle of Anglesey	2.4	-2.2	0
Isle Of Wight	3.2	-2.5	-1
Kent	9.8	-8.1	-2.3
Lancashire	-28.1	26.5	-2.1
Leicestershire	13.4	-6.8	-13.6
Lincolnshire	6.9	-5.8	-1.2
Merseyside	-49.6	61.1	-39.6
Merthyr Tydfil	-3.8	-1.2	11.7
Midlothian	-22.5	0.6	50.2
Monmouthshire	4.7	-2.3	-5.1
Moray	1.1	-2.5	3.8
Neath Port Talbot	-6.5	-8.9	37.5
Newport	-12.2	0.5	26.8
Norfolk	5.2	-5.2	1.3
North Ayrshire	-1.6	-2.5	9.9
North Lanarkshire	-30.2	-20.5	120.9

North Yorkshire	9.8	2.6	-29.2
Northamptonshire	6.4	-4.9	-2.4
Northumberland	-23.8	19.6	5.3
Nottinghamshire	6.9	8.8	-38
Orkney Islands	1.6	-1.2	-0.5
Oxfordshire	75.5	-56.2	-32.4
Pembrokeshire	3.2	-3.3	0.7
Perth & Kinross	1.1	-2.8	4.4
Powys	2.4	-2.6	1
Renfrewshire	-15.1	-9.9	59.7
Rhondda, Cynon, Taff	-16.4	-12.7	69.7
Rutland UA	2.3	-1.6	-1.3
Scottish Borders	1.4	-1.9	1.7
Shetland Islands	1.3	-1	-0.4
Shropshire	2.3	-3.3	3
Somerset	24.1	-8.5	-34.2
South Ayrshire	-0.4	-1.3	4.1
South Lanarkshire	-32.9	-21.7	130.3
South Yorkshire	-38.6	50.4	-38
Staffordshire	-45.6	-12.5	136.3
Stirling	-4	-2.7	16
Suffolk	4.4	-3.8	-0.5
Surrey	65.2	-50.8	-22.2
Swansea	-11.7	-19.1	74.9
The Vale of Glamorgan	-8.9	-8.9	42.8
Torfaen	-2.2	-1.8	9.7
Tyne And Wear	-60.2	60.5	-13.8
Warwickshire	2.5	0.7	-7.4
West Dunbartonshire	-12.9	-7.5	48.6
West Lothian	-22.4	2.3	45.8
West Midlands	5.5	9.4	-36.3
West Sussex	6.6	-5.5	-1.4
West Yorkshire	-38.4	56.2	-52.9
Wiltshire	15.3	-10.4	-8.9

Wrexham	-7.3	-6.9	34

Table S8: Parameters and command lines for benchmarking different IBD detection tools.

	Parameters	Command Line
GERMLINE (1.5.3)	-min_m 5 -h_extend -haploid	./germline -input ukb_22_200k.vcf.ped ukb_22_200k.vcf.map -output ukb_22_200k_5cm -min_m 5 -h_extend -haploid
RefinedIBD (12Jul18.a0b)	length=5	java -Xmx200g -jar refined-ibd.12Jul18.a0b.jar gt=ukb_22_200k.vcf out=22_200k map=decLift_HG19_22.map length=5
iLash	slice_size 350<br>step_size 350<br>perm_count 20<br>shingle_size 15<br>shingle_overlap 0<br>bucket_count 5<br>max_thread 20<br>match_threshold 0.99<br>interest_threshold 0.70<br>max_error 0<br>min_length 5<br>auto_slice 1<br>cm_overlap 1<br>minhash_threshold 55	./IBD iLa_22.config
RaPID (1.7)	-w 3 -r 10 -s 2 -d 5	./RaPID_v.1.7 -i 22.vcf.gz -o 22_5 -w 3 -r 10 -s 2 -d 5 -g 22.rMap
